# Quantification of the fractal nature of mycelial aggregation in *Aspergillus niger *submerged cultures

**DOI:** 10.1186/1475-2859-5-5

**Published:** 2006-02-13

**Authors:** Maria Papagianni

**Affiliations:** 1 Department of Hygiene and Technology of Food of Animal Origin, School of Veterinary Medicine, Aristotle University of Thessaloniki. Thessaloniki 54006, Greece

## Abstract

**Background:**

Fractal geometry estimates have proven useful in studying the growth strategies of fungi in response to different environments on soil or on agar substrates, but their use in mycelia grown submerged is still rare. In the present study, the effects of certain important fermentation parameters, such as the spore inoculum level, phosphate and manganese concentrations in the medium, on mycelial morphology of the citric acid producer *Aspergillus niger *were determined by fractal geometry. The value of employing fractal geometry to describe mycelial structures was examined in comparison with information from other descriptors including classic morphological parameters derived from image analysis.

**Results:**

Fractal analysis of distinct morphological forms produced by fermentation conditions that influence fungal morphology and acid production, showed that the two fractal dimensions *D*_BS _(box surface dimension) and *D*_BM _(box mass dimension) are very sensitive indexes, capable of describing morphological differences. The two box-counting methods applied (one applied to the whole mass of the mycelial particles and the other applied to their surface only) enabled evaluation of fractal dimensions for mycelial particles in this analysis in the region of *D*_BS _= 1.20–1.70 and *D*_BM _= 1.20–2.70. The global structure of sufficiently branched mycelia was described by a single fractal dimension *D*, which did not exceed 1.30. Such simple structures are true mass fractals (*D*_BS _= *D*_BM _= *D*) and they could be young mycelia or dispersed forms of growth produced by very dense spore inocula (10^8^–10^9 ^spores/ml) or by addition of manganese in the medium. Mycelial clumps and pellets were effectively discriminated by fractal analysis. Fractal dimension values were plotted together with classic morphological parameters derived from image analysis for comparisons. Their sensitivity to treatment was analogous to the sensitivity of classic morphological parameters suggesting that they could be equally used as morphological descriptors.

**Conclusion:**

Starting from a spore, the mycelium develops as a mass fractal and, depending on culture conditions, it either turns to a surface fractal or remains a mass fractal. Since fractal dimensions give a measure of the degree of complexity and the mass filling properties of an object, it may be possible that a large number of morphological parameters which contribute to the overall complexity of the particles, could be replaced by these indexes effectively.

## Background

In submerged culture the morphology of filamentous microorganisms varies between two extreme forms, pellets and free filaments, depending on culture conditions and the genotype of the applied strain. A close link between mycelial morphology and productivity has early been identified in important industrial processes such as the citric acid fermentation and several antibiotic fermentations [1]. There has been lots of discussion over the two extreme forms and control of mycelial morphology in fermentations is often a prerequisite for industrial application. The application of image analysis systems in the 1990s permitted the extraction of quantified information and the detailed characterization of various morphological forms [2]. Since then, the relationship between morphology and productivity has been described quantitatively [3], as well as the effect of mechanical and physicochemical parameters on macro- or micro-morphology [4,5], and various structured models have been constructed using important morphological parameters [6]. Morphological parameters most often used, include: the main hyphal length, total hyphal length, number of tips, branching frequency, and the hyphal growth unit for individual mycelial trees, while the area, perimeter, compactness, roughness, circularity and many others for aggregated mycelial particles (pellets and clumps). Although a large number of parameters have been introduced, irregular structures of mycelial particles and structures lying between the two extreme forms of pellets and free filaments, are still difficult to be described by these parameters alone.

Describing mycelia quantitatively essentially involves estimating their space-filling capacity. Density is inappropriate because the number of units of length, volume or area identified, varies with scale of observation. By averaging the heterogeneities of mycelial cultures, density estimates lose functionally important local details that may be valuable in comparing physiological states, e.g. heterogeneities regarding the percentage volume of vacuoles in hyphae are important when estimating the volume of metabolically active mycelium [7-9]. Like many naturally irregular structures, mycelia are approximately fractal [10]. They are amenable therefore, to fractal geometry and the fractal dimension can be used to quantify the extent to which mycelia permeate space in relation to extent of the system. This can be achieved most easily using image capture and analysis techniques.

The fractal concept was proposed by Mandelbrot [11] as a means of describing dimensions "between" the conventional dimensions of 1, 2, and 3 and structures that are neither Euclidean lines and surfaces, nor solids. Fractal dimension spans Euclidean dimension in that it indicates the degree to which an image or object outline deviates from smoothness and regularity: for example a fractal dimension from 1 to 2 describes the area filling capacity of a convoluted line; a fractal dimension between 2 and 3 describes the volume filling capacity of a highly rugged surface. In familiar Euclidean objects like spheres or cubes, constant proportionalities exist among linear dimension (i.e. radius or side length), surface area, and volume, given by

length ∝ surface area^1/2 ^∝ volume ^1/3 ^    Eqn [1]

For highly convoluted structures and images, such relationships are governed by different exponents that are derived from the object's fractal geometry. A feature of mathematically constructed fractal objects is self-similarity, the attribute of having the same appearance at all magnifications or length scales. Fractals, self-similarities, have been applied to describe natural phenomena such as deposition of inorganic material, shape of seashore, cloud formation, diffusion in porous materials [12]. It has also been applied in particle morphology studies [13], in food structure studies [14], and in environmental studies [10,15]. In biological systems, fractals were introduced to describe growth patterns and morphology [16].

The fractal nature of mycelia has been studied [10,16] at two distinct levels using the measures of the surface/border fractal dimension (*D*_BS_), effectively allowing discrimination between systems which are only fractal at their boundaries having entirely plane-filled interiors, and the mass fractal dimension (*D*_BM_), applicable in cases where the interior of the system has gaps (a measure of the space-filling capacity of the interior). Thus, estimates of both mass fractal dimension (which can be considered as a descriptor comparing actual area covered with area enclosed within the minimum perimeter which could contain the whole system) and border fractal dimension (which can be considered as a descriptor comparing the sum of perimeters within the system with its minimum perimeter) are appropriate. Such estimates have proven useful in studying the growth strategies of fungi in response to different environments on soil or on agar substrates, using mostly the "box-counting" method, but their use in mycelia grown submerged is still rare. In view of the above, a study on the effects of certain important fermentation parameters has been undertaken, such as spore inoculum level, phosphate and manganese concentrations in the medium, on mycelial morphology of the citric acid producer filamentous fungus *Aspergillus niger*, as determined by fractal geometry. The value of employing fractal geometry to describe mycelial particles is examined by comparison with information from other descriptors including the classic morphological parameters derived from digital image analysis.

## Results

**Figure 1:**
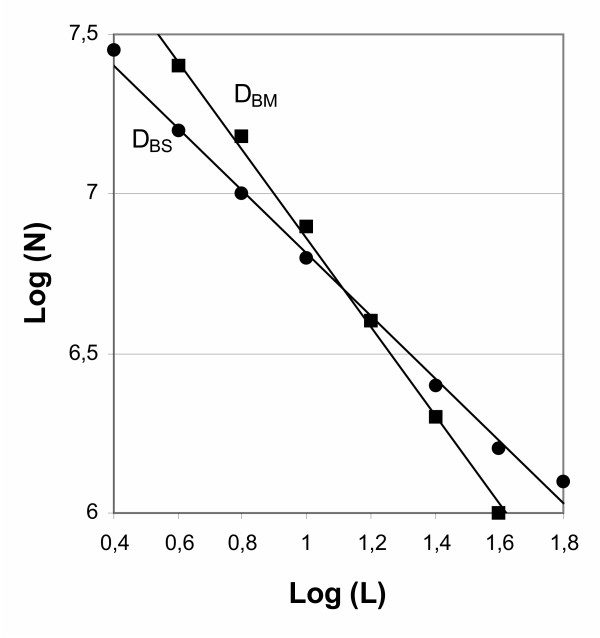
Relationship between experimental values of number of boxes (*N*) *vs *box length (*L*) determined by the BS and BM methods.

**Figure 2:**
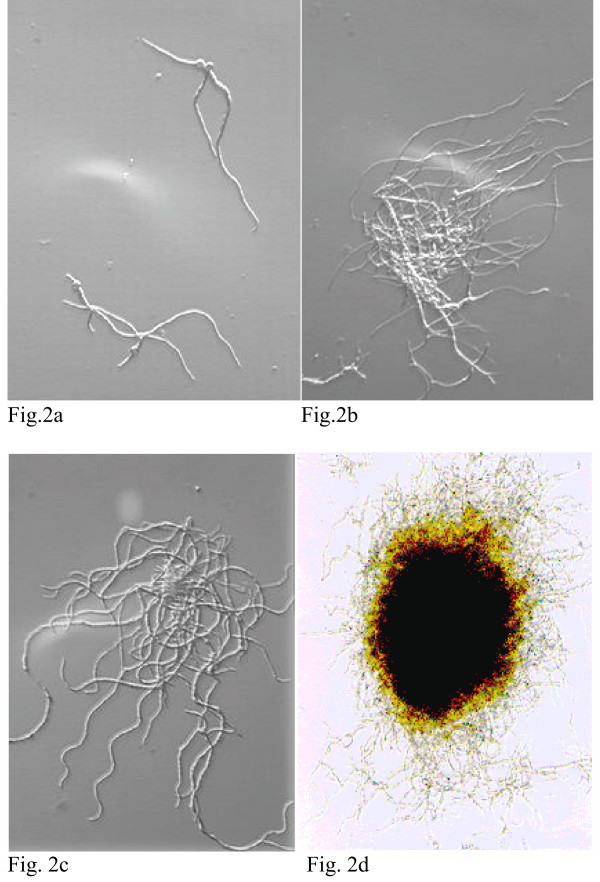
Typical morphologies of *A. niger *mycelium in submerged cultures: (a) 20 hours-old mycelium, (b) a clump, (c) pellet, (d) pellet.

According to Equation [3] (see the Materials and Methods section), a log-log plot of overlapped box numbers *vs *box length should be linear within upper (*L*max) and lower (*L*min) length scale limits (Fig. 1). Any fractal object is an object in which similar structural patterns are repeated at different length scales so as small sections of the object upon magnification appear very similar to the original object. This self-similarity holds with changing scale over a wide range but breaks down beyond finite cutoff values at low as well as high length scales. In this work, the mean diameter of a mycelial particle of 5 μm was applied to the lower length scale limit of self-similarity. Half of the smaller side length of a captured image, typically 25–70 μm, was applied to the upper limit of length scale. Figure 1 shows an evaluation of mycelia in morphological forms ranging from a simple mycelial tree to a clump and finally to a pellet (Fig. 2) and the experimental values of number of boxes (log values) *vs *box length (log values) determined by the BS (•) and BM (■) methods. Below the lower length scale limit and above the upper limit of length scale, numbers of boxes (*N*) overlapped by the mycelium were scattered severely and biased from linear relationship.

**Figure 3:**
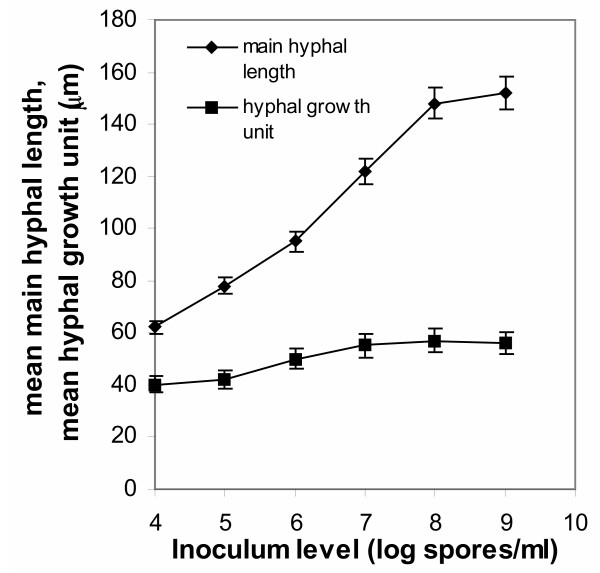
The relationship between the main hyphal length and the hyphal growth unit of dispersed mycelial forms and the spore inoculum level.

**Figure 4:**
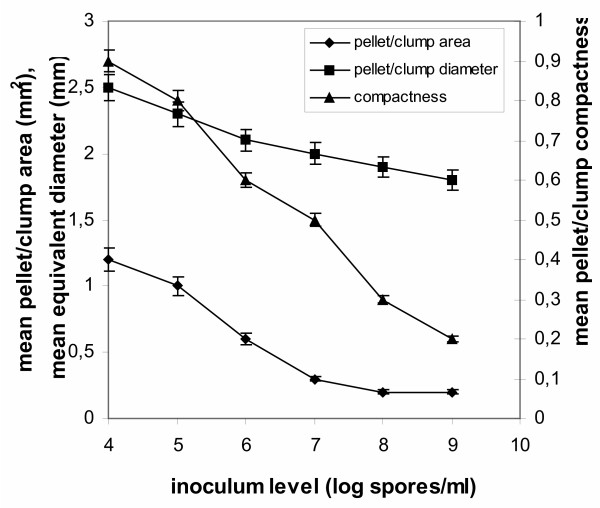
Mean values of area, equivalent diameter and compactness of mycelial aggregates at spore inoculum concentrations ranging from 10^4 ^to 10^9 ^spores/ml.

The main effects of spore inoculum level on *A. niger *morphology are presented in Figs 3 and 4. Since in all cases of tested spore inoculum levels, various morphological forms were identified apart from the dominating form, in Figs 3 and 4 all levels of tested inocula were plotted against morphological parameters corresponding to free filamentous or aggregated material. Fig. 3 shows the effect on the dispersed forms of growth (filamentous morphologies), where a big increase in the mean main hyphal length is observed as inoculum levels increase from 10^4 ^to 10^9 ^spores/ml. The mean total hyphal length and the number of tips per mycelium similarly increase (not shown), a fact reflected in the calculated mean hyphal growth unit (Fig. 3). Fig. 4 shows the effect of varying spore inoculum concentrations on the aggregated forms (clumps and pellets), where the mean projected area occupied by a clump or pellet and the mean equivalent diameter of the aggregated particles decrease with increasing inoculum level. Characteristic is the effect on the compactness of the aggregates. These develop in compact forms at low inoculum levels (10^4 ^to 10^5 ^spores/ml). Compactness reduces and the mycelium develops mainly in the form of clumps as inoculum levels increase towards 10^7 ^spores/ml. Beyond that level the main form of growth is the free filamentous form with single mycelial trees. Productivity was high in this set of experiments and citric acid concentration in the broth reached or exceeded 100 g/l by the end of fermentations at 150 hours, in all cases (not shown). However, citric acid production in fermentations performed with inocula of 10^6 ^and 10^7 ^spores/ml reached 120 g/l, while 110 and 103 g/l obtained from fermentations inoculated with 10^4 ^and 10^5 ^spores/ml, respectively. Filamentous morphologies obtained from fermentations performed with inocula of 10^8 ^and 10^9 ^spores/ml gave in both cases 100 g/l citric acid.

**Figure 5:**
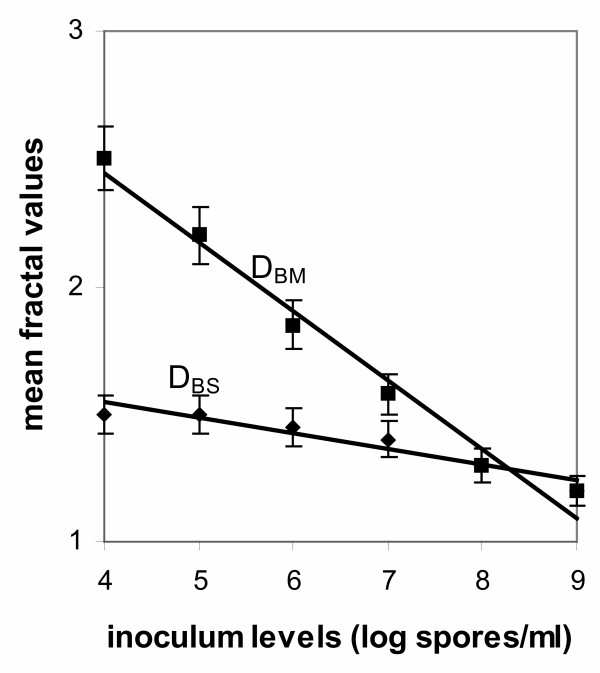
Mean values of fractal dimensions *D*_BS _and *D*_BM _of mycelial particles from fermentations inoculated with spore inoculum concentrations ranging from 10^4 ^to 10^9 ^spores/ml (70 hours samples).

Fractal analysis of the range of morphological forms obtained at 70 hours samples in fermentations inoculated with spore inocula ranging from 10^4 ^to 10^9 ^spores/ml is given in Table 1 and Fig. 5. Table 1 gives the analysis of fractal curves for standard objects, such as a line, a square and a circle for reference and examples of mycelia in the free filamentous form, as well as mycelia aggregated in the forms of clumps and pellets. Further in plotting the fractal values obtained from the two box-counting methods against spore inoculum levels there is obviously an area in which the two dimensions *D*_BS _and *D*_BM _become almost indifferent (Fig. 5) with values close to 1.00. In this case, mycelial particles are mass fractals and this is the case of free filamentous morphologies obtained at inoculum levels of 10^8 ^and 10^9 ^spores/ml. In all other cases, fractal dimensions decreased with increasing spore inoculum concentration.

**Table 1:** Fractal analysis of standard objects and typical morphologies of *A. niger *mycelium

Analyzed object or mycelial particle	*L*^a^	Mean ± SD^b^	
		*D*_BS_	*D*_BM_
Object			
Line	512	1.004 ± 0.019	1.004 ± 0.019
Square	424	1.002 ± 0.009	1.993 ± 0.027
Circle	349	1.000 ± 0.012	1.999 ± 0.011
Mycelial aggregates (typical morphologies)			
Mycelial tree	0.04	1.27 ± 0.02	1.29 ± 0.02
Clump	0.50	1.44 ± 0.02	1.52 ± 0.02
Pellet	0.30	1.45 ± 0.02	1.90 ± 0.02

^a ^*L *is expressed in pixels for objects and millimeters for mycelial aggregates

^b ^Standard deviations were obtained by regression analysis

**Figure 6:**
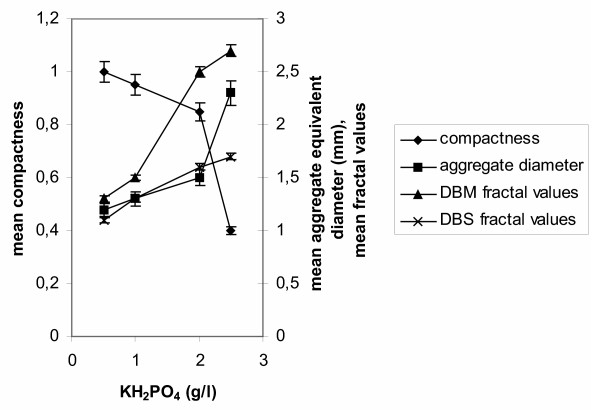
The effect of KH_2_PO_4 _concentration on *A. niger *morphology. Compactness, equivalent diameter and fractal dimensions of mycelial aggregates at KH_2_PO_4 _concentrations ranging from 0.5 to 2.5 g/l.

The effect of phosphate concentration in the medium on fungal morphology is shown in Fig. 6. Increasing phosphate concentration, resulted in increased aggregate diameters. The form of the aggregates was strongly influenced and the main morphological form at 2.50 g/l KH_2_PO_4 _was that of large mycelial clumps. This change in the structure of the aggregates is shown in Fig. 6 where the mean compactness of mycelial structures is plotted against tested phosphate concentrations. Mean values of the fractal dimensions of aggregates increased with increasing phosphate concentrations and the effect was more pronounced on the mass fractal dimension *D*_BM _according to Fig. 6, which increased from 1.30 to 2.30. In this figure, the *D*_BM _line shows a trend similar to that of aggregate equivalent diameters. The surface fractal dimension *D*_BS _ranged between 1.22 and 1.50 for KH_2_PO_4 _concentrations ranging from 1.00 to 2.50 g/l, respectively. Morphological changes were not the only effect of varying phosphate concentrations. Comparing the runs over 150 hours of fermentation, we observed that the maximum specific rate of citric acid production obtained with 2.50 g/l KH_2_PO_4 _was 0.10 h^-1 ^as compared with 0.25 h^-1 ^in the standard run (not shown). At 150 hours, the citric acid concentration in the fermentation broth was 110 g/l in the standard run performed with 2.00 g/l KH_2_PO_4_, while 52 g/l in the 2.50 g/l KH_2_PO_4 _run, 85 g/l with 1.00 g/l KH_2_PO_4 _and 60 g/l with 0.50 g/l KH_2_PO_4_. Biomass increased by almost 20% in the 2.50 g/l KH_2_PO_4 _run compared with the standard run. When concentrations below 2.00 g/l KH_2_PO_4 _were used, biomass levels were lower. The standard medium used in this study is a known production medium for citric acid producer *A. niger *and the particular phosphate concentration of 2.00 g/l KH_2_PO_4 _is the optimum concentration. Below and above the standard phosphate concentration citric acid production reduces.

**Figure 7:**
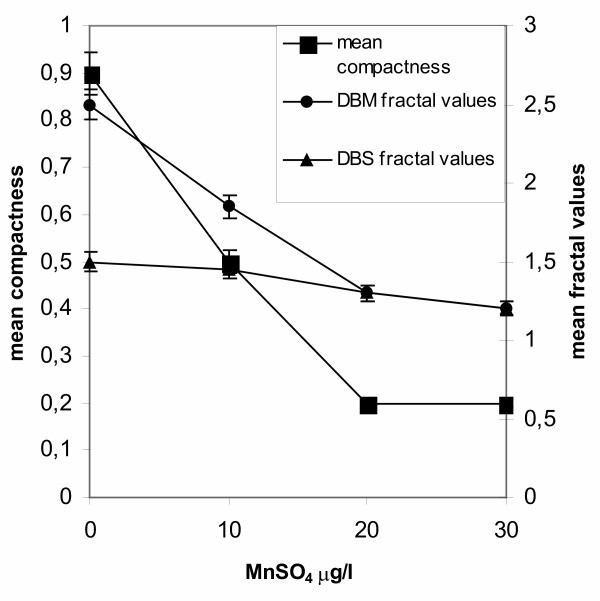
The effect of MnSO_4 _concentration on *A. niger *morphology: mean compactness of detected objects and fractal dimensions at MnSO_4 _concentrations ranging from 0 to 30 μg/l.

In manganese free medium the applied inoculum of 10^4 ^spores/ml resulted in the formation of pellets as shown in Fig. 3. Addition of as little as 10 μg/l MnSO_4 _in the medium resulted in the development of mycelial clumps and a severe reduction in fermentation rates. Citric acid concentration determined at 150 hours found to be reduced by 45% compared to the standard run (not shown). Further increasing the MnSO_4_concentration to 20 and 30 μg/l MnSO_4_, prevented the mycelium from clumping and morphology turned exclusively to the free filamentous form. Citric acid production fell dramatically to as low concentrations as 10 g/l by the end of both runs. Fig. 7 shows the profile of the compactness of mycelial particles and the fractal dimensions values of the mycelium cultivated at various manganese levels. The mean aggregate compactness plot shows a sharp drop with increasing manganese concentration levels. Mean values of both fractal dimensions follow the same trend. *D*_BM _and *D*_BS _for pellets, obtained in manganese free media, approximated 2.52 and 1.50, respectively. For clumps, produced with addition of 10 μg/l MnSO_4 _in the medium, *D*_BM _and *D*_BS _values reduced as 1.85 and 1.45, respectively. Standard deviations of means did not exceed a 5% in all cases. Filamentous morphologies gave *D*_BM _and *D*_BS _values similar to those obtained from filamentous morphologies produced at high inoculum levels (Fig. 5) and as with the inoculum case the measured values for *D*_BM _and *D*_BS _were almost identical.

## Discussion

The mycelium in Fig. 2a grows in *d *= 2, whereas the mycelium in Figs 2b, 2c and 2d grows in three-dimensional space. The fractal evaluation of the aggregate (pellet) in Fig. 2d gives *D*_BS _= 1.45 ± 0.01 and *D*_BM _= 1.95 ± 0.02. This *D*_BM _value is close to 2.00 something we would expect for a planar photographic projection for such a surface fractal. In this aggregate, hyphae grow and fill the area completely in the center of the object. Hyphae also grow at the surface and this growth is characterized by the box surface dimension. In the case of Fig. 2b, the evaluation of the aggregate (mycelial clump) gives a *D*_BM _value reduced to 1.50 ± 0.02, while the box surface dimension is *D*_BS _= 1.44 ± 0.02. The filamentous mycelium in Fig. 2a gives *D*_BM _= 1.29 ± 0.01 and *D*_BS _= 1.27 ± 0.01. This result implies that the structure of Fig. 2a is a self-similar mass fractal over the range of *L *values plotted in Fig. 1. The analysis of deterministic fractal curves by the BM and BS methods shows that *L*max is found in the range *L*/3.5 to *L*/4.5 and this is according to the methods described by Farin et al. [17] and Obert et al. [16]. The quality of the standard deviations of the *D*_BS _and *D*_BM _values from the regression analysis should not be overestimated if the box-counting methods are performed with a 512 × 512 pixel resolution image analysis system as the one used in the case of the present study. To detect the range of the real error, we followed the method of algorithmically constructed self-similar curves according to Farin et al. [17]. For such fractal curves it is possible to calculate *D *analytically and experimentally by the BM and BS methods. For the test objects presented in Table 1 the deviations fall in the range -0.01<*D*- *D*_BM _<0.04 and 0<*D*- *D*_BS_<0.04 and this range is valid only if the standard deviation of *D*_BM _or *D*_BS _obtained by regression analysis is less than or close to 0.02 (Table 1). This is in agreement with results presented by Farin et al. [17] and Obert et al. [16].

The fractal values relate the morphology fundamentally to the distribution of hyphal mass in space. Measurements of fractal dimensions have been more commonly used to provide a quantitative description of changes in the shape of developing colonies of fungi and actinomycetes [18,19]. Box counting techniques showed that the fungus *Sordaria macrospora *grew only as a mass fractal, where box mass and box surface dimensions were equal [16], but the fungus *Ashbya gossypii *showed a transition from mass fractal during early growth to a surface fractal at later stages of colony development where values for box surface and box mass dimensions differ [16] due to overlap of growing hyphae at the center of the colony (that is, only the edge is fractal). That work suggested that the fractal dimension is an important morphological measurement of microbial growth and it is capable of detecting different growth behavior. Fractal analysis has been performed mainly on cultures growing on solid surfaces. On such surfaces, fungi and actinomycetes usually grow as a complex branching mycelium producing round colonies with filamentous edges. The shape of the colonies of filamentous microorganisms has been successfully assessed using box-counting techniques [10,19]. Also various methods of *D *determination have been reported and computer programs that allow box counting of images have been evaluated [20]. In all cases reported so far, *D *values obtained for colonies of filamentous microorganisms confirmed that fractal geometry is valuable for describing differences between colonies.

In submerged culture filamentous microorganisms can grow in a large number of distinct morphological forms, varying between the extremes of pellets and free filaments. The exploitation of image analysis in fungal biotechnology during the last decade permitted quantitative characterization of mycelial structures during important fermentation processes and investigations on the relationship between morphology and productivity of commercially important metabolites like antibiotics, enzymes, and organic acids [1]. Moreover, it permitted construction of structured models [6] that relate morphology to production and helped in understanding the complex rheology of filamentous fermentation broths [21]. A large number of morphological parameters have been introduced [2] in order to give detailed descriptions of mycelial structures and artificial neuronic networks have been applied to distinguish the various morphological forms [22].

Using image analysis implies that many numbers are absolutely necessary in the process of quantitative characterization of mycelial structures. However, in the work of Patankar et al. [23], a new technique, based on a fractal model, was reported for the quantification of the microscopic morphology of mycelia in submerged fermentations. The morphological structuring was treated as a fractal object, and the fractal dimension determined by an ultrasonic scattering procedure developed for the purpose. The fractal model yielded a single, quantitative index of morphology and in experiments with three different species (*Penicillium chrysogenum*, *Streptomyces tendae *and *S. griseus*), the fractal dimensions of pelleted structures were found to be in the range of 1.45 – 2.00, while those of filamentous structures were in the range of 1.90–2.70, with values around 2.00, representing mixed morphologies. In that work no discrimination was made between mass fractal and border fractal dimensions, instead a single fractal dimension *D *was used which showed strong correlation with the index of cake compressibility and with the Kozeny constant, two filtration parameters that are known to be morphology dependent. A single fractal dimension was also used in the work of Lejeune and Baron [24] but on simulated tri-dimensional mycelial structures of fractal nature. There is one more case in the literature, the report by Ryoo [25], in which a fractal dimension was used to characterize pelleted morphologies of *A. niger*. Since only pellets were considered, the single fractal dimension used should correspond to the box surface dimension. The fractal dimension was compared with conventional morphological parameters derived from image analysis studies.

In the present work two box-counting methods were used, yielding the *D*_BS _and *D*_BM _fractal dimensions, to evaluate distinct morphological forms of *A. niger *produced by different spore inoculum concentrations, and different phosphate and manganese levels in the medium. Among the various parameters which exert a strong effect on the development of fungal morphology in submerged culture, is the spore inoculum level. In a recent work on the effects of spore inoculum levels on *A. niger *morphology [26] we showed that a sharp transition from pelleted to filamentous morphologies takes place as spores' concentration increases from 10^4 ^to 10^9 ^spores/ml. Cell volume fraction analysis at 70 hours showed that inoculation with 10^4 ^spores/ml resulted in pellets that accounted for the 95% of the detected objects. The 10^5^spores/ml inoculum produced a mixture of pellets and clumps, the last accounting for a 15% of the detected objects. Clumps accounted for the 90% of the objects in fermentations inoculated with 10^6 ^and 10^7 ^spores/ml and citric acid production was slightly higher in these two fermentations compared to the others in this set of fermentations. Further increasing the concentration to 10^8 ^and 10^9 ^spores/ml resulted in dispersed morphologies (free filamentous mycelium). The parameter "spore inoculum concentration" proved to be very successful in manipulating fungal morphology and for this reason we chose in the present study to perform fractal analysis on the distinct morphological forms produced at various inoculum levels and categorized by means of an artificial neural network and cluster analysis.

Figs 3 and 4 show the profile of various morphological parameters obtained with image analysis at different inoculum levels. In the dispersed forms of growth, a big increase was observed in the mean main hyphal length, total hyphal length and number of tips as inoculum concentration increases from 10^4 ^to 10^9 ^spores/ml. The effect is mirrored on the calculated mean hyphal growth unit (Fig. 3). The main effects of an increasing inoculum size within the above range of concentrations on the aggregated forms (pellets and clumps) are the decrease of the mean areas occupied by the aggregates and the decrease of the mean equivalent diameter of the aggregates (Fig. 4). Compactness reduced progressively (Fig. 4) with increasing inoculum level and from compact pellets at 10^4 ^spores/ml inoculum the mycelium develops as free mycelial trees at 10^9 ^spores/ml inoculum. Fractal analysis of standard morphological forms, characteristic (main morphological forms) at given inoculum concentrations, is given in Fig. 5. In this figure, characteristic mean values of *D*_BM _for pellets were 2.20–2.50 and *D*_BS _1.55–1.62. Mean values of fractal dimensions for clumps were for *D*_BM _1.85-1.58 and *D*_BS _1.45-1.40. Free filamentous trees gave mean *D*_BM _values in the range of 1.30-1.21, while *D*_BS _was in the range of 1.30-1.20. In all cases, standard deviations did not exceed a 5% of mean values. Dispersed morphologies obtained in fermentations inoculated with 10^8 ^and 10^9 ^spores/ml were true mass fractals since the two methods gave the same values of *D*_BM _and *D*_BS_. Only in this case, a single fractal dimension *D*, which equals *D*_BM _equals *D*_BS_, can be used. In all other cases objects cannot be described by one dimension but both *D*_BS _and *D*_BM _should be estimated for a full description of surface irregularities and interior filling of an aggregate. Fig. 5 shows that fractal values of the mycelium decrease with increasing spore inoculum level, denoting a decreased tendency of the mycelium to aggregate.

Literature information on the effects of phosphate concentration in the medium of the citric acid fermentation by *A. niger *is rather limited. Most discussion has been around whether phosphate and /or nitrogen limitation are necessary to achieve increased yields [27,28]. Phosphate concentration effects on citric acid producer *A. niger *morphology have been reported earlier by Papagianni [28,29]. In that work, performed with the same strain but in a different reactor type and using different inoculum type (vegetative instead of spore inoculum), three KH_2_PO_4 _concentrations, 0.10, 0.50, and 1.00 g/l of medium, were tested and the morphological parameters of perimeters of clumps, perimeters of the internal cores of clumps, and lengths of protruding filaments were measured. Increasing the concentration of phosphate resulted in larger aggregates (clumps) with a fluffy appearance. The aggregates developed around cores that remained small, compared to the overall size of the aggregate. In the case of the present work we tested four different phosphate concentrations and morphological parameters derived from samples taken at 70 hours appear as in Fig. 6. The standard medium used throughout this study contained 2.00 g/l KH_2_PO_4 _and in that concentration and the particular spore inoculum applied in this series of experiments (10^4 ^spores/ml), the fungus develops in the pelleted form. At lower phosphate concentrations, smaller and more compact pellets were formed, while increasing phosphate concentration to 2.50 g/l KH_2_PO_4 _resulted in the formation of large clumps. Mean values of fractal dimensions for the main morphological forms obtained in each phosphate concentration case are given in Fig. 6. In this plot, the *D*_BS _line follows the mean diameter of pellets for the 3 concentrations of 0.50, 1.00, and 2.00 g/l KH_2_PO_4_. Almost in parallel lies the *D*_BM _values line for the two lower phosphate concentrations. In the case of 2.50 g/l KH_2_PO_4_, along with a sharp drop in compactness and a large increase in aggregate diameters, we observe a large increase in the *D*_BM _value which approximates 2.70. The mean value of box surface fractal dimension *D*_BS _reached 1.70, which was the highest *D*_BS _value obtained. As with the inoculum studies, both fractal dimensions appeared very sensitive in describing the drastic change in the structure of mycelial aggregates. As was expected, 2.50 g/l KH_2_PO_4 _in the medium did not affect fungal morphology only, since effects on biomass and citric acid production were sound. Increased biomass accumulation in that case was associated with significantly reduced acid production levels compared to the standard run performed with 2.00 g/l KH_2_PO_4_. Formation of clumps by increased phosphate levels in this case was associated with much lower specific production rates.

A number of divalent metals have been suggested as being required in limiting amounts for a successful citric acid process and a central role is attributed to Mn^2+ ^[30-32]. Manganese ions are known to be specifically involved in many cellular processes, such as cell wall synthesis, sporulation, and secondary metabolite production. The involvement of Mn^2+ ^deficiency in developing the intracellular conditions that lead to citric acid accumulation have been discussed in detail by Röhr and Kubicek [27]. Concerning *A. niger *morphology, omission of Mn^2+ ^ions from the nutrient medium resulted in abnormal morphogenesis with swelled and bulbous hyphae [33], while addition of trace amounts (2 ppb) changed the morphological form, from pelleted to filamentous [30,32]. In our study we observed a transition from pellets, in manganese free medium, to clumps, when the medium contained 10 μg/l MnSO_4_, and finally to free filamentous mycelium obtained with addition of 20 and 30 μg/l MnSO_4_. Characteristic is the drop in aggregate compactness (plotted in Fig. 7) with increasing manganese concentration, while mean values of fractal dimensions again appear sensitive to morphological changes. In pelleted morphologies, obtained in manganese free medium, mean *D*_BS _values approximate 1.50, while mean *D*_BM _2.52. Both fractal dimensions appeared reduced for clumps obtained with addition of 10 μg MnSO_4 _/l of medium. Mean values of *D*_BS _approximate 1.45 while mean *D*_BM _1.85 (the standard deviation not exceeding a 4% in all cases). Free filamentous trees obtained with addition of 20 and 30 μg/l MnSO_4_, gave mean values for *D*_BM _1.30-1.21 and *D*_BS _1.30-1.20 (the standard deviations not exceeding a 5% of mean values). These were true mass fractals since the two methods gave the same values of *D*_BS _and *D*_BM _and these are the same with the values obtained from filamentous morphologies in fermentations performed in manganese free media inoculated with 10^8 ^and 10^9 ^spores/ml.

Morphological differences of mycelial structures correlate with pathogenicity [34], metabolic activity [1], enzyme production [1,35-37]. Mycelia have been described as pellets, clumps, and free filamentous, while aggregated structures have been characterized as diffuse, compact, hairy, fluffy, smooth, rough, etc [38]. Image analysis applications in fungal biotechnology have been invaluable in characterizing precisely the various forms with morphological parameters eliminating the confusion of verbal characterizations. Because of the complex structure of mycelia, a geometric, pattern-orientated description, which leads to a measure of irregularity, was impossible without the development of fractal geometry. Fractal geometry has made important contributions to understanding a plethora of natural phenomena. It has been used to describe the growth of inorganic systems in such processes as aggregation, cluster formation, and dendritic growth [39,40]. In biology, fractal geometry has been applied to describe the branching system in the lung airways [41], the brain cortex [42], primary cancer [43], the structure of proteins and the irregularity of their surface [44,45] and mitochondrial genomes [46] among many others. In mycology, it has been used successfully to describe colony growth. In the present case, fractal analysis performed on morphologically distinct forms of *A. niger *in the citric acid fermentation, produced by external factors such as inoculum size or varying concentrations of particular medium constituents, showed that the fractal dimensions are very sensitive in describing such forms and therefore, they can serve as important morphological characteristics of mycelial growth in submerged systems. By applying the two box-counting methods, we have shown that sufficiently branched mycelial particles are self similar, e.g. young mycelia of 20 hours (Fig. 2a), or the dispersed morphological types obtained in fermentations inoculated with 10^8 ^and 10^9 ^spores/ml and those obtained in fermentations performed with 20 and 30 μg MnSO4. This property implies that the global structure of an object may be complex, although the fundamental growth concept -in other words, the way to generate the complex system- may be very simple [11]. Fractal analysis in this study has shown that starting from a spore, the mycelium develops as a mass fractal and, depending on external conditions, it either turns progressively to a surface fractal or remains a true mass fractal. Since filamentous morphologies obtained from very dense spore inocula or elevated manganese levels in the medium gave identical fractal dimensions values we cannot argue that a direct correlation of fractal values to productivities is feasible. However, this obviously holds when comparisons are being made within the same set of experiments. It is important to emphasize that fractal dimension is a generic term without strict definition and it covers a number of different and related measures. Fractal analysis should always be used cautiously and related to the object being measured. Smith and co workers [47] suggest that in neuron cells, which bear some resemblance to growing colonies of branching filamentous microorganisms, the ruggedness of the border (that is rugged, jagged, or uneven borders), the amount of branching, and the space filling properties, that may reflect the extent of branching, contribute to the complexity, with an increase in each contributing to an increase in *D*.

## Conclusion

The research described in this work, shows that sensitivity to treatment effects is a major benefit of using fractal dimension (determined by box-counting) as a descriptor. Information provided from complementary descriptors derived by image analysis can be used in combination when a full picture of the mycelium is required. In any case, fractal analysis provides quantitative indexes of morphology that may have general applicability and can be used in empirical or theoretical correlations between morphology and other properties. The attraction of fractal analysis lies in its ability to quantify aspects of development of a growing fungus, and doing so providing us with a new tool to objectively quantify the effect of various changes in the culture environment.

## Materials and methods

### Microorganism, inoculum preparation, medium

An industrial strain of *Aspergillus niger *(*A. niger *PM1) was used throughout this work. This was maintained on molasses agar, which contained 300 g/l cane molasses (pH adjusted at 6.8), and 18 g/l agar (Technical, Grade 3, Oxoid, Basingstoke, U.K.). The agar plates were incubated at 30°C for 7 days. Spores were collected from mature culture plates and spore suspensions were diluted with sterile medium to make a range of concentrations in the order of 10^4 ^spores /ml to 10^9 ^spores /ml of media.

The composition of the standard fermentation medium was the following (g/l): D-Glucose, 150.00; (NH_4_)_2_SO_4_, 2.50; MgSO4.7H_2_O, 0.50; KH_2_PO_4_, 2.00; Fe^3+ ^[as Fe_2_(SO)_4_.24 H_2_O], 0.10 × 10^-3^; Zn^2+ ^[as ZnSO_4_.7 H_2_O], 0.10 × 10^-3^; Cu^2+ ^[as CuSO_4_.5 H_2_O], 0.06 × 10^-3^. Three additional KH_2_PO_4 _concentrations in the medium were also tested, 0.50, 1.00 and 2.50 g/l, while the effect of manganese ions on fungal morphology was examined in manganese free media and in media containing 10, 20 and 30 μg/l MnSO_4_. In studies on the effect of phosphate and manganese concentrations on *A. niger *morphology the spore inoculum concentration applied was 10^4 ^spores /ml of media.

### Analytical methods

Dry weights were determined by filtering 20 ml of broth through pre-weighed glass fiber filters (grade GF/C, 4.25 cm, Whatman International, Maidstone, U.K.), washing and drying in a microwave oven (15 min at low power) and left in a dessicator for 24 hours before reweighing. Citric acid was determined by the method of Marier and Boulet [48].

### Culture conditions

The stirred tank bioreactor used in this work was a 3.0 L New Brunswick Scientific BIOFLO 110. The reactor was equipped with baffles. The agitation system consisted of two 6-bladded Rushton-type impellers (52 mm), operating at a stirrer speed of 400 rpm. Process temperature was maintained at 28°C and the air-flow rate at 1 vol/vol/min air/medium (vvm). pH was controlled at 2.1 by the automatic addition of titrants (2 M NaOH and 20% H_2_SO_4 _solutions). Polyethylene glycol (M.W. 2000, Sigma) was used as antifoam in all fermentations. Fermentations terminated at 150 hours from inoculation.

### Image analysis and processing

Fungal morphology was characterized by using a semi-automatic image analysis system consisting of an Olympus microscope (Olympus, New Hyde Park, NY, U.S.A.) operated as phase contrast, a CCD camera (Sony, Cambridge, U.K.), a PC with a frame-grabber, and the image analysis software (SIS, Olympus, Germany). Samples preparation and measurements were as described in earlier publications [5,49,50]. Morphological features evaluated by image analysis were classified using an artificial neural network (ANN) [26]. The ANN considered four main object types, globular and elongated pellets, clumps and free mycelial trees. The significance of morphological features and their combination was determined by cluster analysis [26]. Monitoring of mycelial morphology throughout a large number of *A. niger *fermentations revealed the well-known pattern of fragmentation and re-growth, described in detail in an earlier report [9]. Studies on the errors of object identification, by means of the ANN, showed that the period in which morphology is established and it appears rather stable before fragmentation takes place, expands from 50 to 90 hours and therefore the timing of 70 hours was chosen for morphological characterization of fungal particles. At that time also we avoid growth-related problems in fractal analysis. Spores, following germination, produce a straight or a zigzag hypha, which under fractal analysis should yield a *D *value close to 1. Later, branching occurs and fractal behavior is naturally associated with hyphal branching. In young mycelia there is not usually a sufficient number of branches to allow for a well-defined power law behavior. The morphological parameters evaluated, included: the main hyphal length, total hyphal length, number of tips, branching frequency and the hyphal growth unit for individual mycelial trees, as well as the area, perimeter, equivalent diameter, circularity, eccentricity, compactness and roughness for mycelial aggregates (clumps and pellets). Equivalent diameter is the diameter of a circle having the same area with the measured feature. It is derived from area (A) as . Compactness (known also as fullness) is a measure of the voidage of a particle and is used to characterize mycelial clump and pellet structures. It is the ratio of the actual area of the particle to the convex area. For a smooth pellet (without hairy regions) compactness approximates 1, while for a loose clump it is less than 1. A full list of parameters obtained by image analysis and definitions were given by Paul and Thomas in their review on characterization of mycelial morphology using image analysis [2]. Fermentations were carried out in triplicates. For morphology measurements, an average of 500 objects were measured per sample. Morphological data are presented as mean values.

### Fractal analysis

The box-counting method described by Obert et al. [16] and Donelly et al. [19] were used to determine the fractal dimensions of the mycelial systems. Fractal analysis and calculation of mycelial area were carried out using the Image-J 1.33 software that is available in the public domain via the NIH [51]. Following filtering all unwanted material and correcting optical errors, areas were calculated on binary images by counting the number of black pixels in each image and multiplying this by the area represented by each pixel. When the mycelium is covered by a grid of equal side length (*L*), the number of boxes (*N*) overlapped by the mycelium could be counted. The number of boxes overlapped by a mycelium image grows as the side length *L *of box is increased. For a series of boxes of side length *L *pixels, the number of boxes intersected by the set (*N*) is related to the fractal dimension of the set (*D*) by the power law

*N*(*L*) = α*L*^*D *^    Eqn [2]

where α, is a proportionality constant [52]. Equation [2] can be expressed in logarithmic form as

log *N*(*L*) = *D*log*L *+ logα     Eqn [3]

Interior boxes, which are contained wholly within the fractal set, and border boxes, which contain or adjoin at least one black pixel, contribute to the total number of boxes (*N*) intersected by the set. Thus,

*N*(*L*) = *N*_border _(*L*) + *N*_interior _(*L*)     Eqn [4]

Mycelial structures can be mass fractals, where the whole mass of the particle is fractal, or surface fractals, where the surface (border) only is fractal [16]. To distinguish between these two kinds of fractals, two different box-counting methods were applied according to Obert et al [16]. The box mass (BM) method is applied to the whole mass of the mycelium, which leads to the box mass dimension, *D*_BM_. In the box surface (BS) method, only those boxes that cover the surface (border) of the mycelial particle have to be counted. The BS method leads to the box surface dimension, *D*_BS_. The box-counting evaluations of digitized structures are performed by Image-J. An estimate of the border fractal dimension is obtained by plotting log *N*_border _(*L*) against log*L*. Regression analysis of the linear portion of this plot yields a gradient of -*D*_BS_. The mass fractal dimension is similarly estimated by regression analysis on the linear portion of the plot of log [*N*(*L*) - 1/2 *N*_border _(*L*)] against log*L*, yielding a gradient of -*D*_BM_. To measure the border fractal dimension, the outline function of Image-J was used. This eliminates all black pixels except those that form the margin or border of the mycelium. In the case in which a mycelial structure is a mass fractal, the two methods give the same values of *D*_BM _and *D*_BS_. In this case, the fractal dimension *D *is equal to *D*_BM_, which equals *D*_BS_. For a surface fractal, the object cannot be described by one single dimension *D*. The *D*_BS _value in this case describes the surface irregularities and it is equal to the fractal dimension *D*. The *D*_BM _value describes the dimension of the filling space *d*. In this analysis, the *d *value equals 2, based on the analysis of photographs which are planar projections of objects that grow in *d *= 2 or *d *= 3. More than 100 mycelia from samples corresponding to 70 hours of fermentations were processed and the means of fractal values were derived at various culture conditions. Both box-counting methods were performed with a 512 × 512 pixel resolution image analysis system. For most cases, coefficients of determination (R^2^) were more than 0.95 representing good linear correlation.

## List of abbreviations and symbols

A, area

ANN, artificial neural network

BM, box mass method

BS, box surface method

*d*, filling space

*D*, fractal dimension

*D*_BM_, mass fractal dimension

*D*_BS_, surface (border) fractal dimension

*L*, side length of boxes

*N*, number of boxes

α, proportionality constant
